# Regional biomechanical imaging of liver cancer cells

**DOI:** 10.7150/jca.32985

**Published:** 2019-07-25

**Authors:** Weiwei Pei, Jiayao Chen, Chao Wang, Suhao Qiu, Jianfeng Zeng, Mingyuan Gao, Bin Zhou, Dan Li, Michael S. Sacks, Lin Han, Hong Shan, Wentao Hu, Yuan Feng, Guangming Zhou

**Affiliations:** 1 State Key Laboratory of Radiation Medicine and Protection, School of Radiation Medicine and Protection, Collaborative Innovation Center of Radiological Medicine of Jiangsu Higher Education Institutions, Soochow University, Suzhou 215123, China; 2 Center for Molecular Imaging and Nuclear Medicine, School of Radiological and Interdisciplinary Sciences (RAD-X), Soochow University, Collaborative Innovation Center of Radiation Medicine of Jiangsu Higher Education Institutions, Suzhou 215123, China; 3 Guangdong Provincial Engineering Research Center of Molecular Imaging, the Fifth Affiliated Hospital, Sun Yat-sen University, Zhuhai 519000, China; 4 School of Biomedical Engineering, Science and Health Systems, Drexel University, Philadelphia, PA 19104, USA; 5 Institute for Medical Imaging Technology, School of Biomedical Engineering, Shanghai Jiao Tong University, Shanghai 200240, China; 6 Willerson Center for Cardiovascular Modeling and Simulation, Institute for Computational Engineering & Sciences, the University of Texas at Austin, TX 78712, USA

**Keywords:** biomechanics, AFM, indentation, liver cancer, cell stiffness

## Abstract

Liver cancer is one of the leading cancers, especially in developing countries. Understanding the biomechanical properties of the liver cancer cells can not only help to elucidate the mechanisms behind the cancer progression, but also provide important information for diagnosis and treatment. At the cellular level, we used well-established atomic force microscopy (AFM) techniques to characterize the heterogeneity of mechanical properties of two different types of human liver cancer cells and a normal liver cell line. Stiffness maps with a resolution of 128x128 were acquired for each cell. The distributions of the indentation moduli of the cells showed significant differences between cancerous cells and healthy controls. Significantly, the variability was even greater amongst different types of cancerous cells. Fitting of the histogram of the effective moduli using a normal distribution function showed the Bel7402 cells were stiffer than the normal cells while HepG2 cells were softer. Morphological analysis of the cell structures also showed a higher cytoskeleton content among the cancerous cells. Results provided a foundation for applying knowledge of cell stiffness heterogeneity to search for tissue-level, early-stage indicators of liver cancer.

## Introduction

Liver cancer is one of the most fast growing cancer forms in terms of new incidents and death cases 1. It is known that cells can sense mechanical forces and deformations and transduce into biological response 2, 3, 4, 5, 6. Biomechanical properties of cancer cells have shown to be closely related with cancer pathology and metastasis state abnormalities 7, 8. It is also found that the biomechanical changes were correlated with the cell-cell communications 9 and microenvironment 10, 11. Understanding the biomechanical properties of tumor cells can not only help to elucidate the mechanisms behind disease progression, but also provide important information for cancer diagnosis and treatment 12, 13, 14.

Many methods have been used to measure the mechanical properties of individual cells, such as atomic force microscopy (AFM), micropipette aspiration, optical tweezers, etc. 15, 16, 17. Among these methods, AFM-based nanoindentation is one of the most widely used modality to measure the cellular structures 18, 19 and probe the mechanical properties of living cells 20, 21, 22. It has been used to measure micro-scale structures of cells 23, 24, 25, 21 and microorganisms 22, as well as macro-scale structures such as cartilage tissue 26, 27 and brain tissue 28. In this study, we used AFM to characterize the mechanical properties of liver cancer cells. Although many studies have investigated the mechanical properties of tumor cells 15, 21, 24, few studies have investigated the mechanical properties of liver cancer cells. Wu et al. was among the first to investigate the viscoelastic properties of hepatocytes and hepatocellular carcinoma (HCC) cells using micropipette techniques 29. Using AFM nanoindentation, Grady 30 also investigated the elastic properties of the HUH-7 (HCC) cells.

At the tissue level, studies characterizing the mechanical properties of soft tissues have highlighted the heterogeneity of many tissues 31, 32. For example, Plodinec 33 used AFM to characterize the distribution of the mechanical properties at the tissue level as a biomarker for breast cancer diagnosis. At the cellular level, cells have complex surface structures containing a variety of components such as lipid and protein. In addition, beneath the plasma membrane, cytoskeleton of the cell is also a heterogeneous meshwork. Therefore, the mechanical properties of cells are essentially heterogeneous. Guo 34 studied the distributions of elastic parameters to characterize the cell properties. Hecht 35 mapped out the viscoelastic properties of cells with a resolution of 50 × 50 and a pixel resolution of 640-950 nm. For higher resolution mapping of effective modulus, Smolyakov 36 imaged elastic module of bacteria samples with a resolution up to 128×128 for a region of interest about 1.5× 1.5 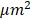. Although differences in mechanical properties between healthy and cancerous tissue have long been studied as biomarkers of various cancers, a feature that has not yet been exploited is mechanical heterogeneity.

In this study, we used AFM nanoindentation method to investigate the biomechanical properties of both liver cancer cells and normal liver cells. Mechanical heterogeneity properties were mapped out with a fine resolution. Distribution features of the effective modulus were compared between the cancerous and normal cells. Results provided biomechanical properties for potential application in liver cancer diagnosis and prognosis.

## Materials and methods

### Cell preparation

Hepatoma cells including Bel7402, HepG2 and human normal liver cell line L02 were purchased from Shanghai Cell Bank of Chinese Academy of Sciences. Cells were cultured in RPMI-1640 medium (Gibco, Grand Island, NY, USA) supplemented with 10% fetal bovine serum (FBS, Gibco, Grand Island, NY, USA), 1% penicillin sodium and 100 μg/mL streptomycin, at 37°C in 5% CO2 in a humidified incubator (Thermo Scientific, Asheville, NC, USA). Normal and hepatoma cell cultures were both maintained in FBS medium under the same conditions. The cells were cultured until tightly adhered to the micropatterned glass-bottom culture dishes. Cells were grown to 10%-20% of their confluency before tests. The 60-mm cell culture dishes were mounted on the sample stage of the AFM. All scanning and measurements were performed on proliferating viable cells maintained to room temperature in Phosphate Buffer Saline (PBS) within 1-2 hours after removal of growth medium and rinse for three times by PBS.

### AFM-nanoindentation

**Figure 1:**
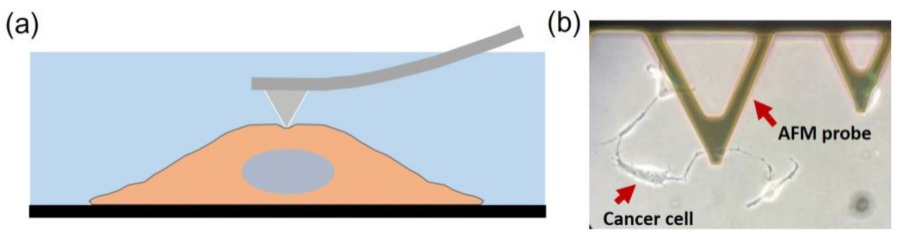
** (a)** Illustration of nanoindentation of cancer cells using AFM. The measurements were carried out with live cells submerged within PBS solution.** (b)** A microscope image showing a typical measurement of liver cancer cell with a MLCT E-type probe.

Indentation tests were performed using a combined atomic microscope with an inverted optical microscope (BioScope Resolve Atomic Force Microscope) at room temperature. Probes with a nominal spring constant of 0.10 N/m are chosen for the experiment (MLCT type E tip, Bruker Co. Ltd., Billerica, MA, USA). We used the Peak Force Quantitative Nanomechanical Mapping in fluid to measure the indentation responses at each testing point. The measurements were carried out with cells submerged within PBS in a liquid testing mode (Figure 1). A field of view (FOV) of 50
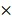
50 μm^2^ was selected for mapping the elastic modulus of the cell with an image resolution of 128x128.

Hertz and Sneddon models were the most used method for characterizing cell properties 25, 37. In this study, the indentation depth is more than 10 times larger than that of the tip radius, therefore the Sneddon model was adopted 34. The four sided pyramid AFM problem was usually modeled by pyramid 38, and the effective modulus could be estimated by


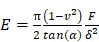
(1)

where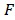 is the indentation force, 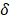 is the corresponding indentation displacement, 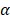 is the semi-included angle of the pyramid tip, and 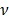 is the Poisson's ratio. We used 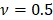 since the cells could be treated as incompressible 14, 34, 38. The modulus map was estimated for each of the 16,384 indentation points using the force-displacement curve acquired. To reduce the substrate effect due to the model assumption of a semi-infinite space, we selected the top region (above the half of the cell height) of the cell covering the nucleus and major cell contents for analysis. This selection guarantees that the thickness of the sample is more than 50 times of the tip radius.

Based on the modulus images, histogram of the probability distribution function was fitted with a normal distribution. The mean value 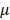 and the standard deviation
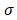
of the distribution with their 95% confidence intervals were estimated for each cell. The average values of 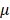 and 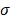 were also compared with respect to the regions acquired from the 6 Bel7402 cells, 6 HepG2 cells, and 6 normal liver cells. To see the differences of effective modulus between each cell type, the measured indentation points for each cell were grouped together for student's t-tests at a significance level of 5%.

### Confocal microscopy

To observe the microstructure of each cell, samples in the glass-bottom dishes were fixed with freshly prepared 4% formaldehyde (Sinopharm Chemical Reagent Co. Ltd.) in PBS for 10 min at room temperature. After fixation, we washed samples with PBS for three times and added 0.1% Triton X-100 (Sigma Aldrich Co. Ltd., Shanghai) in PBS with 1% bovine serum albumin (MesGen, Shanghai Hongsheng biotechnology Co. Ltd) to each for 5 min. After this permeabilization, fluorescein-phalloidin solution (MesGen MF8203, Shanghai Hongsheng Biotechnology Co. Ltd) was applied for 20 min at room temperature inside a covered container during the incubation. The samples were subsequently washed with PBS for three times. We followed the protocol recommended in the product Hoechst 33342 (Sigma Aldrich Co. Ltd, Shanghai) for the cell nucleus staining. Confocal images were taken using a laser scanning confocal microscope (Olympus FV1200, Japan).

## Results

**Figure 2:**
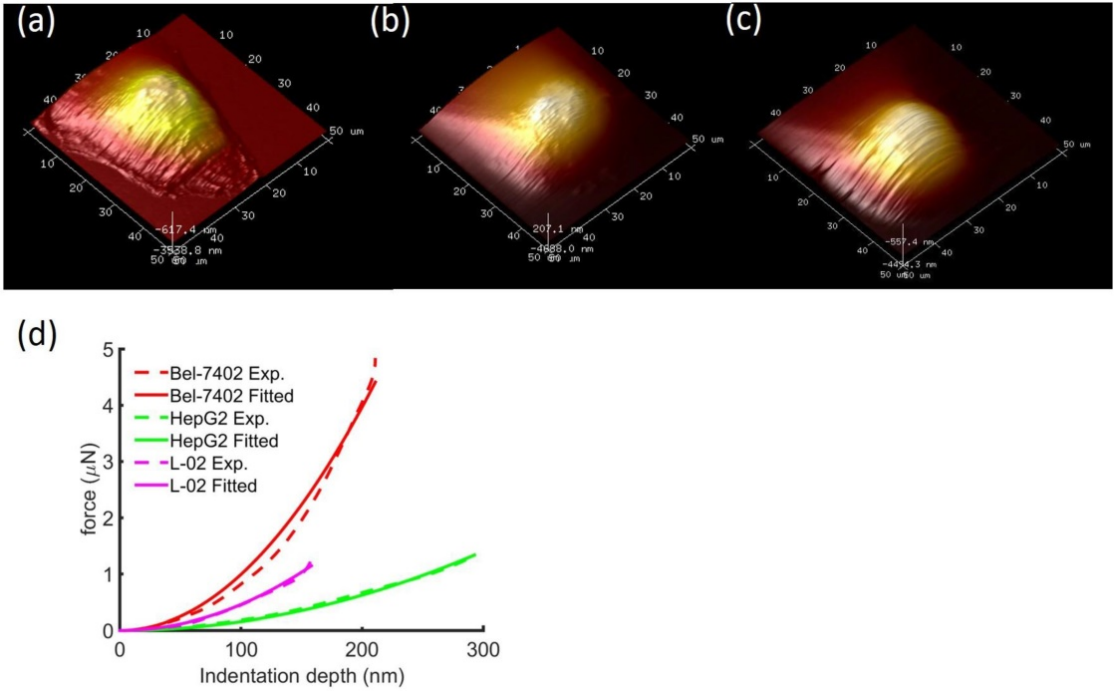
Typical height images of **(a)** Bel7402, **(b)** HepG2, and (c) L02. The image FOV was 50×50μm^2^. Comparison of typical indentation force-displacement curves between the three different cell types **(d)**. The experiment curves were fitted with Sneddon model of Eq. (1). The distinguishable curves of the three cells showed stiffness differences between the cells.

Typical height images of the 3 types of cells showed that cancerous cells were about 2-3 times larger than the normal liver cells (Figure 2). In addition, we also observed the morphological differences between the cell shapes where the cancerous cells have branches of tentacles stretching out of the cell bodies. Typical force-displacement curves of the nanoindentation from different cells showed different effective modulus, where from stiff to soft were Bel7402, L02, and HepG2 cells.

**Figure 3:**
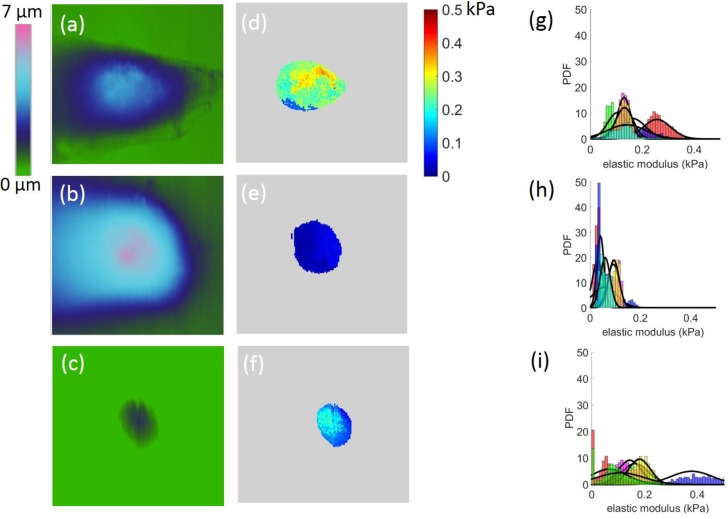
Height images **(a-c)** and the corresponding modulus map **(d-f)** for Bel7402, HepG2, and L02 cells, respectively. The corresponding elastic moduli above the half height of the cells were selected for analysis. The field of view for each image is 50
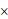
50*μ*m^2^. The estimated probability distribution function (PDF) of the effective modulus distribution for (g) Bel7402, (h) HepG2, and (i) L02 cells. A normal distribution function was fitted to each histogram.

The effective moduli were mapped out with respect to approximately upper 50% height of each cell (Figure 3). We observed that Bel7402 and HepG2 cells had higher moduli than that of L02 cells. Analysis of the probability density function (PDF) of the effective modulus distribution showed significant differences between each cell (Figure 3). For cancerous cells, the mean effective moduli of the Bel7402 were significantly higher than that of HepG2. The L02 cells appeared to have a large standard deviation of effective modulus with a relatively higher value than HepG2 cells. The estimated effective modulus distribution parameters for each cell were summarized in Table 1.The maximum and minimum mean elastic moduli were 0.256 and 0.030 for Bel7402 and HepG2 cells, respectively. The maximum and minimum standard deviation values of the elastic moduli were also observed for Bel7402 and HepG2 cells, which are 0.731 and 0.014, respectively. Ranking from maximum to minimum values of the overall average modulus, we observed an order of Bel7402, L02, and HepG2. One-way ANOVA analysis showed that the mean elastic moduli were significantly different between the three cells but no significant differences were observed for the standard deviation values.

**Figure 4:**
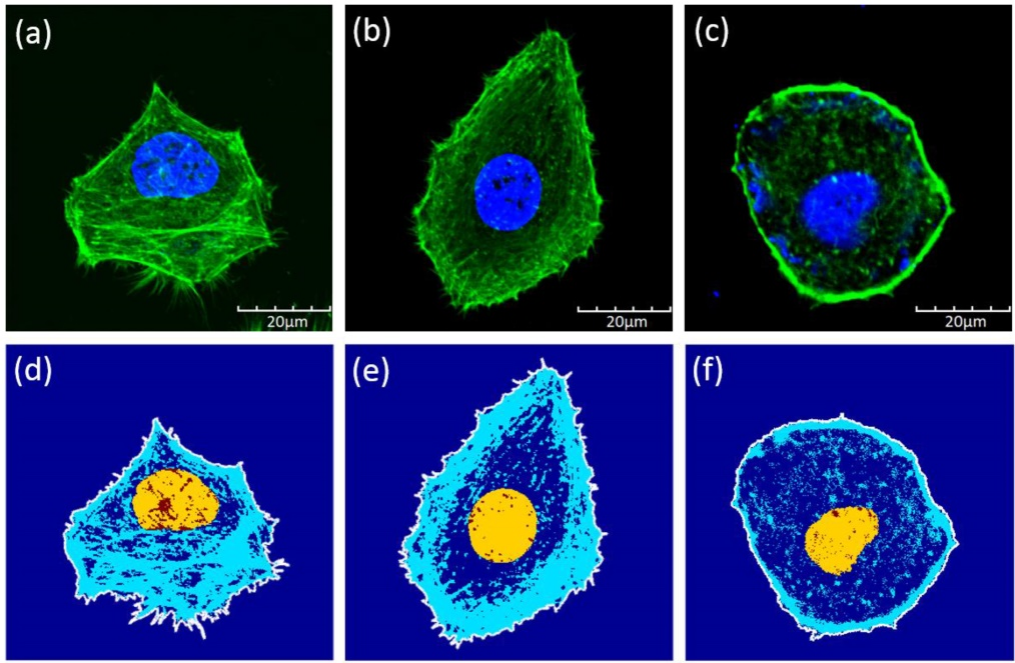
Confocal images of **(a)** Bel7402, **(b)** HepG2, and **(c)** L02 cells. Although the sizes of the cells were similar between each type, the cytoskeleton structures were different among these cells. Illustrations of the cell boundaries (white line), nucleus (yellow region), and the cytoskeleton (blue lines) for **(d)** Bel7402, **(e)** HepG2, and **(f)** L02 cells. The structures were extracted based on the confocal images of Figure 4.

Confocal images of the cells showed that the Bel7402 cells have relatively larger cytoskeleton structures. We analyzed the cytoskeleton ratio of the cells with respect to the whole cell in terms of the areas (Figure 4). The mean ratios were 0.42±0.11, 0.39±0.11, and 0.07±0.03 for Bel7402, HepG2, and L02 cells, respectively. The only significant differences were found between the normal cells and the cancerous cells (student t-test, *p*<0.05).

## Discussion

In this study, we investigated the effective modulus and its heterogeneity of liver cancer cells. Using AFM nanoindentation techniques, we observed that, the cancerous cells and the normal cells had significant different distribution of the effective modulus in the cellular level. Significant differences of the mean modulus were also found between the three types of cells. Analysis of the cellular structure shed lights on the mechanical behaviors.

### Experimental measurement

Many studies have used AFM nanoindentation to investigate the effective modulus of cancerous cells. Some studies have shown that cervical cancer cells (HeLa) have a softer hardness than normal human uterine epithelial cells. Similarly, malignant (MCF-7) breast cells were found to have an apparent Young's modulus significantly lower (1.4-1.8 times) than that of their non-malignant (MCF-10A) counterparts, but limited data were available for liver cancer cells 39, 21. Our study shows that there is no significant difference in the hardness between normal liver cells line and liver tumor cell lines, this may be dependent on different cell lines. In addition, most of investigations focused on the overall mechanical response of the cancerous cells, using a spherical bead indenter or pyramid indenter with respect to selected points on the cell 40. In this study, we provided a detailed mapping of the effective modulus of two different types of liver cancer cells. Unlike cells such as pancreatic beta cells that demonstrated strong adhesion effects during indentation, we did not notice significant adhesion effects for all the cancerous and normal liver cells 41. We found that the effective modulus of the liver cancer cells ranged from 0.03-0.26 kPa, similar to that of the cancerous breast epithelial cells 24. Grady 30 found the median Young's modulus of the HUH-7 cells was 0.3 kPa, which is similar to the effective modulus of Bel7402 cells we measured.

### Influences of morphological structure

It has been known that the cellular structure such as cytoskeleton contributed to the mechanical properties of cells 42. Using gastrointestinal tumor and malaria cells, Suresh 7 found that the effective modulus of cells increased or decreased due to membrane or cytoskeleton reorganization. It was also found that mechanical properties of the cells also depend on the level of cancer transformation. With a low level of transformation, cells were softer than that with a higher level of transformation 43. In this study, we observed that different types of liver cancer cells had significant different effective moduli and the heterogeneous properties. However, by comparing the estimated structure ratio of the cytoskeleton, we found no significant differences between the cancer cells. This indicates that the morphological structure of the cells may not be the main contributor to the mechanical properties of the cells, rather the properties of the structure such as the cytoskeleton or membrane could play a major role.

Sun 44 and Wu 45 founded that differences in cell cytoskeleton (F-actin) were accompanied with changes in the cell migration ability and Young's modulus. Studies have shown that disruption of microtubule dynamics could affect cancer cell effectively 5, 30. Both Grady 30 and Wu 29 found that upon removal of cytoskeleton structures, the elastic properties of the HCC cells decreased. Although we did not notice a significant difference in the volume ratio of cytoskeletons between the cancerous cells, a significant lower composition of cytoskeleton was observed for the normal liver cells. Besides, it has been found that Bel7402 cells have higher migration and invasive capacity than HepG2 cells 46. We postulate that a higher volume ratio of the cytoskeleton structure could contribute to higher migration ability among the tested cancerous cells.

### Correlation between the tissue and cellular level

Using AFM, the measured effective modulus can be used to distinguish normal and cancerous breast cancer cells 24, 47. For most cancerous cells, the effective modulus was lower than the corresponding normal cells 21. Li 24 are among the first to investigate the mechanical properties of breast cancer cells and observed significantly lower apparent Young's modulus of the Malignant (MCF-7) breast cells than that of the non-malignant (MCF-10A) counterparts. We observed that HepG2 was softer than the normal liver cells. However, no significant differences were observed between the Bel7402 and normal liver cells. These type-dependent properties were similar to that of the cervix cancer cells, where CRL2614 cells were stiffer 48 and primary cancer cells were softer 49. Therefore, we showed that cancerous cells were not necessarily softer than normal cells, the hardness of the cells depend on the specific categories and phenotypes.

* In vivo* characterization of the mechanical properties of liver cancer tissue showed that the cancerous tissues were stiffer than that of the normal tissues 50, 51. Similar stiffening behavior in the tissue level was also observed for other cancers such as breast cancer 52. The stiffer behavior at the tissue level were different from that at the cellular level, where we found that only Bel7402 cells were stiffer than the normal liver cells. The differences of the mechanical properties in the cellular and tissue level revealed that besides the mechanical properties of the cells, structures such as extra cellular matrix (ECM) play an important role in the tissue level behavior. In fact, studies have already pointed out that rigid tumors were stiffer than normal tissues because of a stiffer ECM 53. However, cancerous cells that were either stiffer or softer than the normal cells indicated a variation of the roles of ECM in affecting the tissue level mechanics. This provided new clues in understanding the mechanotransduction of cancer cells 53, 54, 55, 56.

### Limitations and future studies

In this study, we only mapped out the effective properties of live cancer cells. However, elastic properties cannot represent the full mechanical characteristics of cancer cells, since viscoelastic behaviors have been observed for many different types of cells 57. Future studies involve finding a way to map out the viscoelastic properties of a live cell within a reasonable time frame. In addition, we will investigate the structural and mechanical properties of the ECM of liver cancer tissues.

## Conclusions

In this study, we used AFM nanoindentation to characterize the mechanical properties of cancerous and normal liver cells. Elastic modulus with a refined resolution were acquired and analyzed. Distinct effective modulus and its distributions were found for each cell type, while significant differences of the effective moduli were found between cell types. The Bel7402 cells had the highest magnitude of effective modulus while HepG2 had the lowest modulus. Although the dispersion of the elastic module was different for each cell, no significant difference was found. Analysis of the morphological structure showed a significantly lower volume ratio of the normal liver cells than the cancerous cells. The results provided helpful hints for understanding the cellular behavior of liver cancer cells as well as the indications for understanding the mechanical differences between cellular and tissue levels.

## Figures and Tables

**Table 1:** Estimated normal distribution parameters for each cell and the corresponding 95% confidence intervals for the parameters. μ and *σ* are the mean and standard deviation values for the normal distribution.

	μ (kPa)	*σ* (kPa)	Average μ (kPa)
Bel7402	0.256±0.003	0.053±0.002	0.154
	0.105±0.001	0.039±8.40x10^-4^	
	0.162±0.003	0.05± 0.002	
	0.130±8.60x10^-4^	0.025±6.09x10^-4^	
	0.131±0.001	0.033±9.80x10^-4^	
	0.139±0.004	0.731±0.002	
HepG2	0.030±9.22x10^-4^	0.021±6.52x10^-4^	0.063
	0.041±7.83x10^-4^	0.014±5.54x10^-4^	
	0.058±9.31x10^-4^	0.049±6.59x10^-4^	
	0.092±0.001	0.023±8.26x10^-4^	
	0.094±8.13x10^-4^	0.021±5.75x10^-4^	
	0.060±9.31x10^-4^	0.020±6.59x10^-4^	
L02	0.165±0.002	0.050±0.002	0.174
	0.379±0.005	0.079±0.003	
	0.144±0.003	0.043±0.002	
	0.181±0.002	0.042±0.002	
	0.067±0.004	0.067±0.003	
	0.109±0.006	0.089±0.004	
